# Enhanced Long-Term Corrosion Resistance of 316L Stainless Steel by Multilayer Amorphous Carbon Coatings

**DOI:** 10.3390/ma17092129

**Published:** 2024-05-01

**Authors:** Shuyu Li, Hao Li, Peng Guo, Xiaowei Li, Wei Yang, Guanshui Ma, Kazuhito Nishimura, Peiling Ke, Aiying Wang

**Affiliations:** 1 Key Laboratory of Advanced Marine Materials, Ningbo Institute of Materials Technology and Engineering, Chinese Academy of Sciences, Ningbo 315201, China; lishuyu@nimte.ac.cn (S.L.); lh@nimte.ac.cn (H.L.); lixw0826@gmail.com (X.L.); yangwei1991@nimte.ac.cn (W.Y.); maguanshui@nimte.ac.cn (G.M.); kazuhitonishimura@nimte.ac.cn (K.N.); kepl@nimte.ac.cn (P.K.); 2 Center of Materials Science and Optoelectronics Engineering, University of Chinese Academy of Sciences, Beijing 100049, China; 3 School of Materials Science and Physics, China University of Mining and Technology, Xuzhou 221116, China

**Keywords:** corrosion, salt spray, long-term, multilayer DLC, stainless steel

## Abstract

Diamond-like carbon (DLC) coatings are effective in protecting the key components of marine equipment and can greatly improve their short-term performance (1.5~4.5 h). However, the lack of investigation into their long-term (more than 200 h) performance cannot meet the service life requirements of marine equipment. Here, three multilayered DLC coatings, namely Ti/DLC, TiC_x_/DLC, and Ti-TiC_x_/DLC, were prepared, and their long-term corrosion resistance was investigated. Results showed that the corrosion current density of all DLC coatings was reduced by 1–2 orders of magnitude compared with bare 316L stainless steel (316Lss). Moreover, under long-term (63 days) immersion in a 3.5 wt.% NaCl solution, all DLC coatings could provide excellent long-term corrosion protection for 316Lss, and Ti-TiC_x_/DLC depicted the best corrosion resistance; the polarization resistances remained at ~3.0 × 10^7^ Ω·cm^2^ after immersion for 63 days, with more interfaces to hinder the penetration of the corrosive media. Meanwhile, during neutral salt spray (3000 h), the corrosion resistance of Ti/DLC and TiC_x_/DLC coatings showed a certain degree of improvement because the insoluble corrosion products at the defects blocked the subsequent corrosion. This study can provide a route to designing amorphous carbon protective coatings for long-term marine applications in different environments.

## 1. Introduction

With the rapid advancement of the ocean economy, an increasing amount of marine engineering equipment is being produced and utilized for ocean measurement, observation, and forecasting. Meanwhile, in seawater, certain essential components of marine equipment, such as manipulators and environmental monitoring systems, encounter a range of challenges, such as scratch and corrosion problems [1,2,3,4,5]. Surface and coating technology are effective methods to extend the service life of those crucial components and realize their reliable operation without altering the fundamental characteristics of the substrate materials [6,7,8,9].

Among various coatings, diamond-like carbon (DLC) coatings are extensively used as scratch-resistant and anti-corrosion coatings for metal materials due to their high hardness and excellent chemical stability [10,11,12,13,14]. Recent efforts have seen a surge in the exploration of various DLC coatings for application in marine environments, showcasing enhanced performance over short durations [15,16]. For example, Totolin et al. found that the W-doped DLC coatings could effectively improve the tribocorrosion performance of the substrates in artificial seawater within 4.5 h [17]. Wei et al. confirmed that the incorporation of a multilayer structure can endow DLC coatings with improved anti-corrosion protection for 0.5 h. However, the presence of microdefects was identified as a pivotal constraint limiting their electrochemical corrosion resistance [18]. In light of the potential mechanical wear and tear experienced in marine settings, Li et al. designed a Cr/DLC multilayered film with a thickened top layer, which exhibited outstanding corrosion and tribocorrosion resistance in artificial seawater over a testing period of 1.5 h [19].

Meanwhile, in consideration of the long-term immersion conditions in a marine corrosive environment, DLC coatings encounter numerous challenges. Maguire et al. found that Si-doped DLC coatings significantly improved the corrosion resistance of the substrates, but the adhesion strength of the coatings decreased by 75% following a one-month immersion in bodily fluids [20]. Turcio-Ortega et al. delved into the corrosion resistance of DLC coatings immersed in a 0.89 wt.% NaCl solution for 3–195 h [21]. Their findings revealed that as the immersion time increased, the corrosive solution gradually penetrated the pores of the coatings, reaching the interface and triggering local corrosion, thus compromising the protective performance against corrosion.

Generally speaking, the short-term protection provided by DLC, lasting between 1.5 and 4.5 h, appears satisfactory. The incorporation of multiple interfaces has proven to be an effective strategy for enhancing the anti-corrosion and tribocorrosion resistance of DLC. However, research on its long-term performance, exceeding 200 h, is lacking. The underlying degradation mechanism remains ambiguous, failing to meet the durability requirements of marine equipment.

In addition, our prior research found that the failure mechanism of DLC coatings under different hydrostatic pressure corrosion environments was different, while the damage tolerance of amorphous carbon coatings during long-term friction corrosion processes has also been confirmed [22,23,24]. For example, our team developed a sandwich-structured amorphous coating with a Ti/TiC_x_ transition layer on the surface of 316L stainless steel. This coating demonstrated remarkable long-lasting anti-tribocorrosion capabilities in artificial seawater surroundings and under a 10 N load, enduring for 12 h and covering a distance of 1728 m. It was observed that the wear and corrosion damage tolerance of the coating was attributed to the formation of graphitized amorphous carbon and oxide nanocrystals at the interface [25]. Nonetheless, the enduring corrosion-induced damage to the coating remains inadequately explored. Hence, an examination of the long-term corrosion performance of multilayer DLC coatings under complex working conditions will facilitate the development of DLC coatings with adaptability across multiple environments in the future.

As an extension of the preliminary work [25], in this study, three kinds of DLC coatings with various interface structures, namely Ti/DLC, TiC_x_/DLC, and Ti-TiC_x_/DLC, were prepared on 316L stainless steel (316Lss) by compositing a linear anode-liner ion source (LIS) and direct-current magnetron sputtering (DCMS). The electrochemical corrosion resistance of the three kinds of DLC-coated substrate and bare 316Lss was compared in a 3.5 wt.% NaCl solution. Furthermore, both electrochemical impedance spectroscopy (EIS) tests after long-term (63-day) in-situ immersion and neutral salt spray (3000 h) were used to evaluate their long-term corrosion resistance. The role of interface structures in the protective performance of DLC was also discussed.

## 2. Materials and Methods

**Figure 1:**
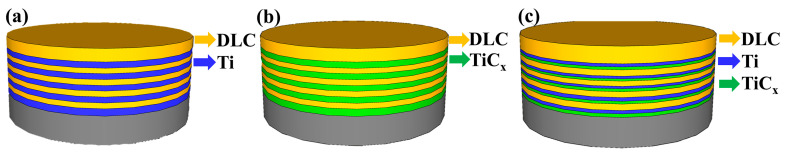
The structure of (**a**) Ti/DLC, (**b**) TiC_x_/DLC, and (**c**) Ti-TiC_x_/DLC multilayer coatings.

Three kinds of multilayer DLC coatings with alternating Ti layers (TiC_x_ layer and TiC_x_-Ti-TiC_x_ layer) and DLC layers were prepared; their thicknesses were kept at around 1.4 μm, as shown in Figure 1. Compared with Ti/DLC and TiC_x_/DLC coatings, Ti-TiC_x_/DLC coatings had more interfaces, and all the neighboring two layers (Fe-Cr/Ti, Ti/TiC_x_, and TiC_x_/DLC) were close in thermal expansion coefficient and mechanical properties, which was expected to endow the Ti-TiC_x_/DLC with both high adhesion and excellent corrosion resistance [26,27,28].

**Figure 2:**
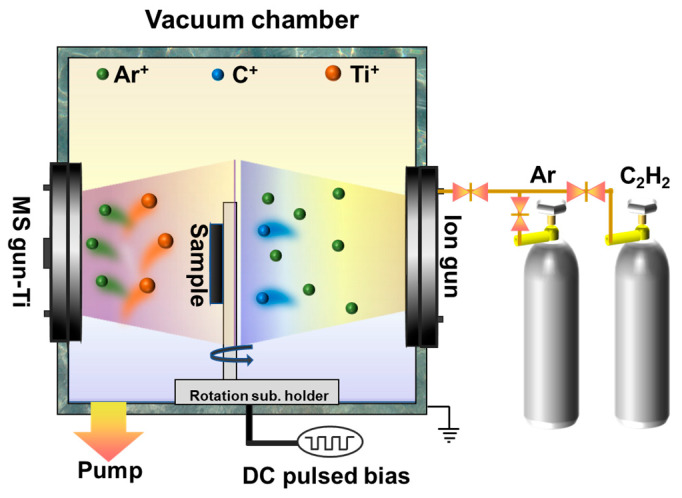
Schematic diagram of the hybrid deposition system.

Three coatings were deposited both on silicon P (100) wafers and 316Lss using a hybrid ion beam deposition system. This equipment consisted of one LIS supplied with C_2_H_2_ gas for DLC deposition and one DCMS unit provided with a pure Ti target (>99.99%), as shown in Figure 2. After ultrasonic cleaning in alcohol for 15 min and drying in air, the substrates were put into the vacuum chamber. Prior to deposition, the chamber was evacuated to about 2.0 × 10^−5^ Torr and heated to 150 °C. Then the substrates were etched using Ar^+^ bombardment for 30 min at a bias voltage of −200 V to remove the surface impurities. More details on sample preparation can be found in our previous work [19,29].

The surface morphologies of the DLC coatings were characterized by scanning electron microscopy (SEM; Thermo Scientific Verios G4 UC, Waltham, MA, USA) equipped with an energy-dispersive spectrometer (EDS; Oxford X-Max, Oxford, UK). The cross-section morphologies were observed with a transmission electron microscope (TEM, FEI Tecnai F20, Hillsboro, OR, USA). X-ray photoelectron spectroscopy (XPS; Shimadzu Axis ultraDLD, Kyoto, Japan) was utilized to characterize the chemical composition and bonds of the DLC coatings. Using monochromatic Al (mono) Kα irradiation at a pass energy of 160 eV, the analyzed area was cleaned by Ar^+^ ions at 2 keV for 5 min before measurement. Here, the sp^2^ content and sp^2^/sp^3^ ratio in the DLC were determined from the obtained C 1s core-level spectra calibrated at 284.6 eV, using a mixture of a Gaussian (20%) and a Lorentzian (80%) fitting after a Shirley background subtraction by CasaXPS software (2.2.73) [30,31,32]. The microstructure and atomic bond of the coatings were analyzed by Raman spectroscopy (Renishaw inVia-reflex, London, UK) with a laser wavelength of 532 nm and an acquisition time of 10 s. The mechanical properties, including hardness and elastic modulus, were tested by the continuous load-controlled nano-indentator (MTS NANO200, Eden Prairie, MN, USA). The electrochemical measurements were performed on a ModuLab (Solartron Analytical, San Diego, CA, USA) electrochemical workstation by a conventional three-electrode electrochemical cell, which contained samples with an exposed area of 1 cm^2^ serving as the working electrode, a saturated calomel working as the reference electrode, and a platinum plate as the counter electrode. Experiments were carried out at room temperature in a 3.5 wt.% NaCl solution. All potentials in this work were measured using a saturated calomel electrode (SCE). Potentiodynamic measurements were initiated after a steady open circuit potential (OCP). The potential was from −0.4 to 1.5 V at a sweep rate of 0.5 mV/s. The EIS technique was used to evaluate the corrosion properties of samples before, during, and after corrosion. The EIS measurements were registered at OCP in the frequency range of 10^5^ to 10^−2^ Hz with a 10 mV sinusoidal perturbation. The acquired data were analyzed using Modulab (2.1.5121) and Zview (3.1) software. For comparison, the corresponding EIS measurements were also conducted on the 316Lss.

## 3. Results

### 3.1. Microstructure Characterization

**Figure 3:**
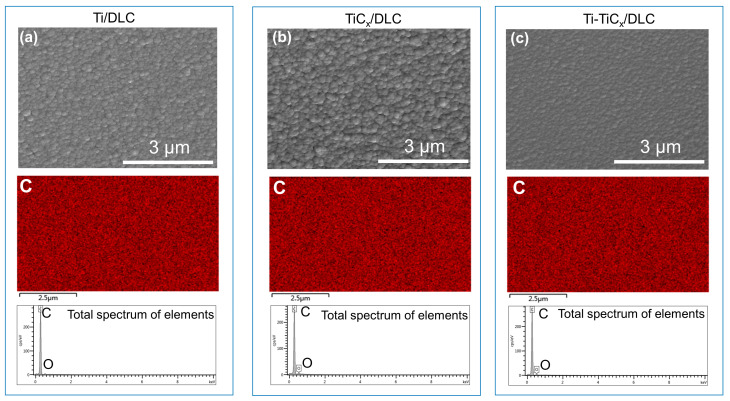
The surface SEM images and EDS of (**a**) Ti/DLC, (**b**) TiC_x_/DLC, and (**c**) Ti-TiC_x_/DLC multilayer coatings (C is carbon; O is oxygen).

Figure 3 shows the surface SEM images of Ti/DLC, TiC_x_/DLC, and Ti-TiC_x_/DLC. All the coatings showed continuous, uniform, and smooth surfaces without visible defects. The roughness of the coatings decreased in the order of Ti/DLC, Ti-TiC_x_/DLC, and TiC_x_/DLC, which was mainly related to the different growth characteristics of the Ti layer and TiC_x_ layer. The distribution of elements corresponding to each coating showed that the surface of the coatings was predominantly composed of carbon elements with a small amount of adsorbed oxygen. No signals for titanium were detected due to a thicker top a-C layer (200 nm) in the top layer, resulting in a weaker signal in the bottom layer.

**Figure 4:**
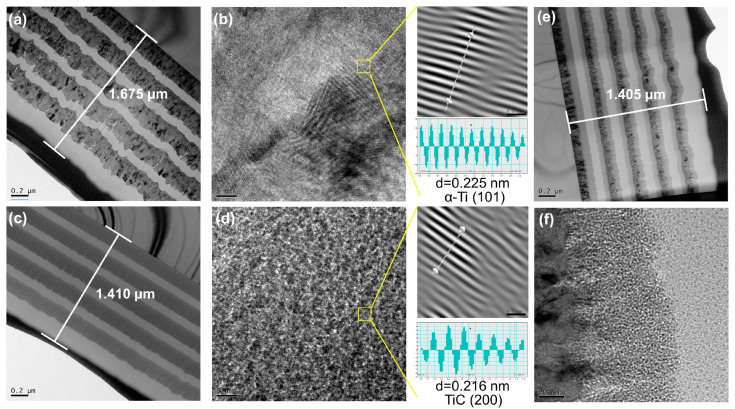
The cross-section TEM images of (**a**) Ti/DLC, (**b**) the enlarged view of Ti/DLC, (**c**) TiC_x_/DLC, (**d**) the enlarged view of TiC_x_/DLC, (**e**) Ti-TiC_x_/DLC, and (**f**) the enlarged view of Ti-TiC_x_/DLC.

Figure 4a,c,e portrays the TEM images showcasing the cross-sections of Ti/DLC, TiC_x_/DLC, and Ti-TiC_x_/DLC, respectively. The thickness of these three coatings ranged from 1.4 to 1.7 μm, presenting a clear multi-layered composition. In Figure 4b, an amplified perspective of the Ti layer and lattice spacing within Ti/DLC are revealed. It is evident that the Ti layer showed a crystalline arrangement with a lattice spacing measuring 0.225 nm. Compared to PDF cards, the predominant composition of the Ti layer appeared to be Ti (100). Turning to Figure 4d, a closer look at the TiC layer within TiC_x_/DLC featured a structure characterized by amorphous enclosed nanocrystals, exhibiting a lattice spacing of 0.216 nm. Comparative analysis with PDF cards indicated that the primary constituent of the TiC layer was Ti (200). Lastly, Figure 4e,f offers an enhanced view of the Ti-TiC_x_ layer within Ti-TiC_x_/DLC. Notably, the method for preparing the Ti layer mirrored that of Ti/DLC, while the preparation of the TiC layer mirrored that of TiC_x_/DLC. Hence, the structural similarity between them remained apparent.

**Figure 5:**
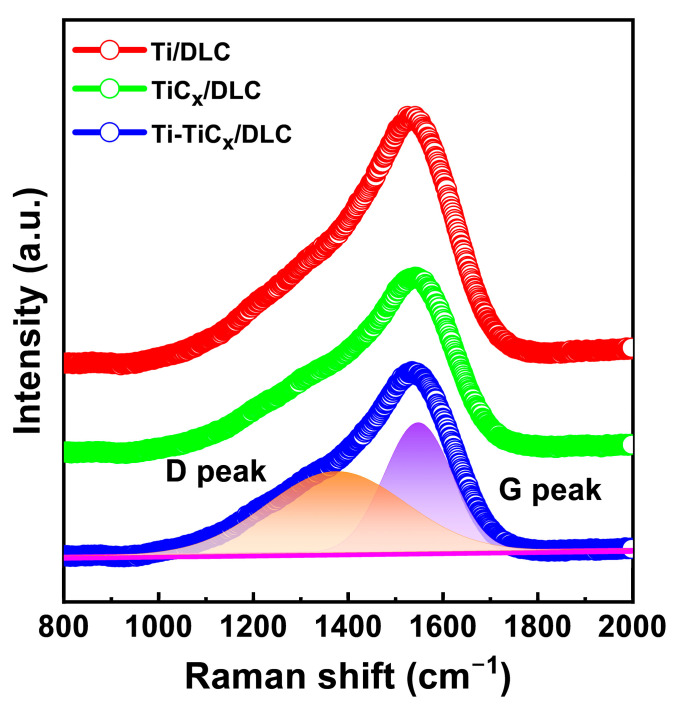
Raman spectra of three kinds of multilayer coatings (The orange background color represents the D peak, while the purple background color represents the G peak).

The atomic bond structure of the DLC coatings was characterized by visible Raman. Figure 5 shows the Raman spectra for clarity. After Gaussian fitting, the spectra could be decomposed into a D peak and a G peak, where the D peak centered at 1360 cm^−1^ originated from the breath mode of sp^2^ bonds and the G peak centered at 1560 cm^−1^ was due to the vibration mode of sp^2^ bonds in both rings and chains. The fitted peak position, peak area ratio I_D_/I_G_, and half maximum of G peak (FWHM) values are summarized in Table 1, which could reflect carbon atom disorder degrees, sp^2^/sp^3^ ratio, sp^2^ cluster size, and so on [33]. The fitting D peak, G peak, G_FWHM_, and I_D_/I_G_ ratio kept stable around 1369 cm^−1^, 1548 cm^−1^, 180 cm^−1^, and 0.57, respectively, which revealed the same atomic bond structure of the top DLC layer.

**Figure 6:**
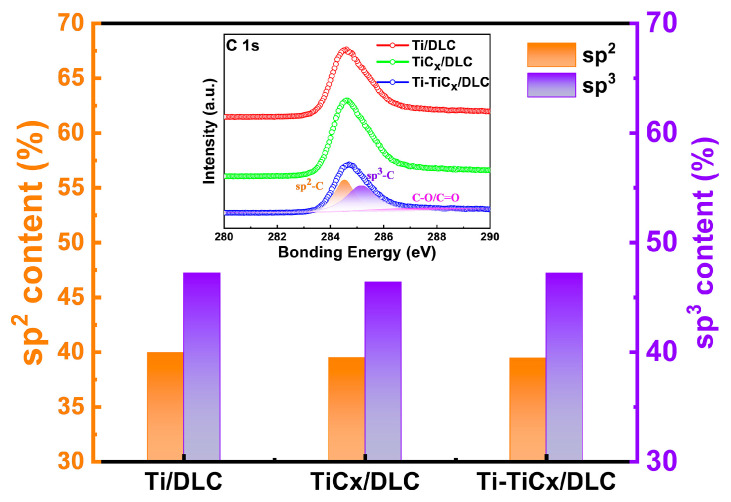
The sp^3^ and sp^2^ contents of three kinds of multilayer coatings. The inset shows the XPS C 1s core-level spectra (The orange background color represents the sp^2^ peak, while the purple background color represents the sp^3^ peak).

Figure 6 shows the XPS C 1s core-level spectra and the fitted results of three kinds of multilayer coatings. The XPS C 1s spectra (inserts in Figure 6) were fitted into three dominant peaks approximately centered at 284.6 eV, 285.4 eV, and 286.6 eV, corresponding to sp^2^, sp^3^, and C-O/C=O hybridization, respectively [31,34,35,36]. The relative contents of sp^3^ and sp^2^-bonded carbon atoms were calculated from the peak area ratio. The sp^2^ and sp^3^ contents of the DLC coatings approximately remained at 39.5 ± 0.5% and 46.8 ± 0.5%, respectively, which agreed well with the Raman result above.

### 3.2. Mechanical Properties

**Figure 7:**
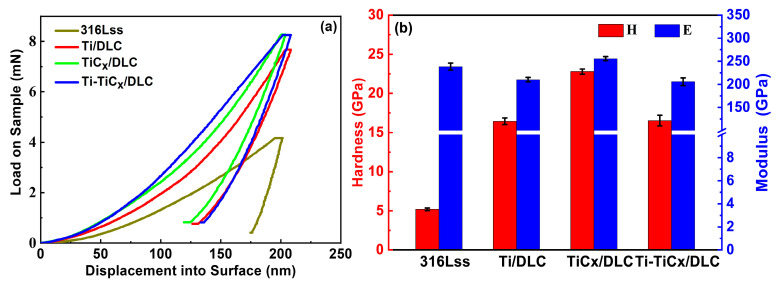
(**a**) Load indentation depth curves; (**b**) hardness and elastic modulus of substrates and three kinds of multilayer coatings.

Nanohardness (H) and elastic modulus (E) of substrates and three kinds of multilayer coatings were measured by the nanoindentation method, as shown in Figure 6. According to the nanoindentation load displacement curve in Figure 7a, the coated samples need a larger load than the substrates at the same indentation depth (200 nm), which reflected an improved hardness of the substrates, and the TiC_x_/DLC had the largest hardness around 22.8 GPa, as depicted in Figure 7b. As frequently mentioned, indexes in nanoindentation analysis, H/E and H^3^/E^2^, were regarded as key parameters reflecting the elastic–plastic deformation properties of materials, which were closely related to fracture toughness and wear resistance. Generally speaking, the higher values of H/E and H^3^/E^2^ mean the higher fracture toughness and wear resistance of the material [37,38,39,40,41,42,43,44]. The calculated H/E and H^3^/E^2^ of 316Lss and the three coatings are summarized in Table 2. Due to the excellent mechanical properties of the deposited DLC, all three coatings could improve the mechanical properties of the 316Lss effectively, and the TiC_x_/DLC coatings showed the highest fracture toughness and wear resistance. The hardness of Ti/DLC was the most minimal, followed by Ti-TiC_x_/DLC, which fell in the intermediate range, and ultimately, TiC_x_/DLC showed the highest level of hardness. This phenomenon may be attributed to the higher hardness of the intermediate layer of TiC in comparison to the Ti layer.

### 3.3. Corrosion Properties

**Figure 8:**
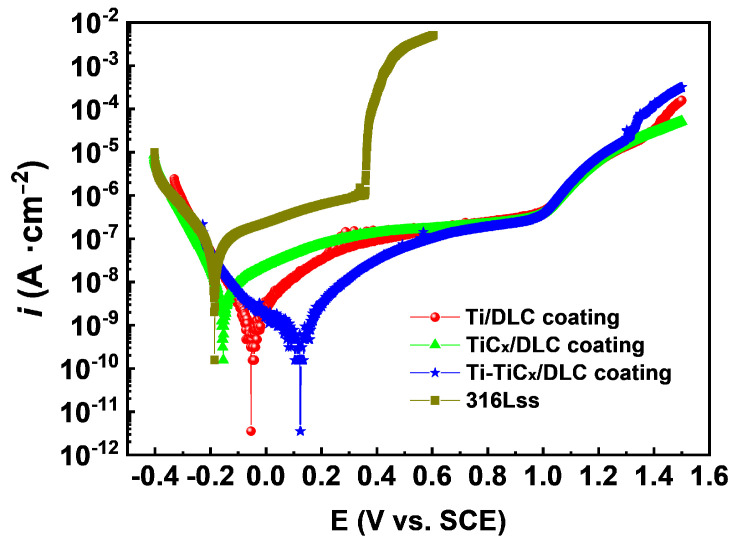
Potentiodynamic polarization curves of three kinds of multilayer coatings and 316Lss in 3.5 wt.% NaCl.

Figure 8 and Table 3 show the potentiodynamic polarization curves and corresponding analysis results of bare 316Lss and coated samples in 3.5 wt.% NaCl. Potentiodynamic polarization curves of all coated samples had more positive corrosion potential (E_corr_), lower passivation current density (i_pass_), and higher pitting potential (E_pit_) compared with 316Lss, which inferred that all the DLC coatings improved the corrosion resistance of substrates significantly [45,46,47]. Especially, the pitting potential of 316Lss increased from 0.36 V to ~1.00 V, which indicated that the pitting resistance of 316Lss was greatly improved in 3.5 wt.% NaCl. In addition, the Ti-TiC_x_/DLC coatings displayed the largest positive E_corr_ of 0.12 V and the lowest i_pass_ of 5.68 × 10^−10^ A/cm^2^ among the three kinds of DLC coatings, which depicted their best corrosion resistance for 316Lss. The polarization resistance R_p_ of Ti-TiC_x_/DLC was around 5.83 × 10^7^ Ω·cm^2^, which was 2 times and 10 times higher than that of Ti/DLC and TiC_x_/DLC, respectively.

**Figure 9:**
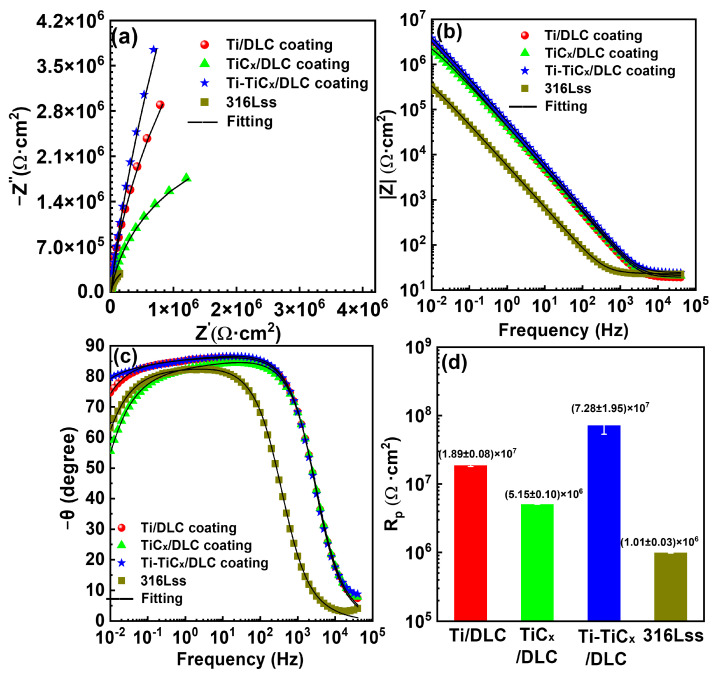
(**a**) Nyquist diagram, (**b**,**c**) Bode diagram, and (**d**) charge transfer resistances of Ti/DLC, TiC_x_/DLC, and Ti-TiC_x_/DLC multilayer coatings and 316Lss in 3.5 wt.% NaCl.

Figure 9 shows the EIS test results of bare substrate and coated 316Lss in 3.5 wt.% NaCl. Compared with bare 316Lss, all coated substrates depicted a larger capacitive arc, a higher low-frequency impedance, and a wider phase angle platform in the Nyquist and Bode diagram, as displayed in Figure 9a–c, which indicated that all the DLC coatings greatly improved the corrosion resistance of 316Lss [48,49]. Ti-TiC_x_/DLC had the largest capacitance–resistance arc radius, indicating a strong ability to hinder charge transfer. Ti-TiC_x_/DLC had a higher phase angle at low frequencies than other materials, representing better corrosion resistance stability and fewer defects. Among the three kinds of DLC coatings, Ti-TiC_x_/DLC exhibited the best corrosion resistance, while TiC_x_/DLC showed the worst performance.

**Figure 10:**
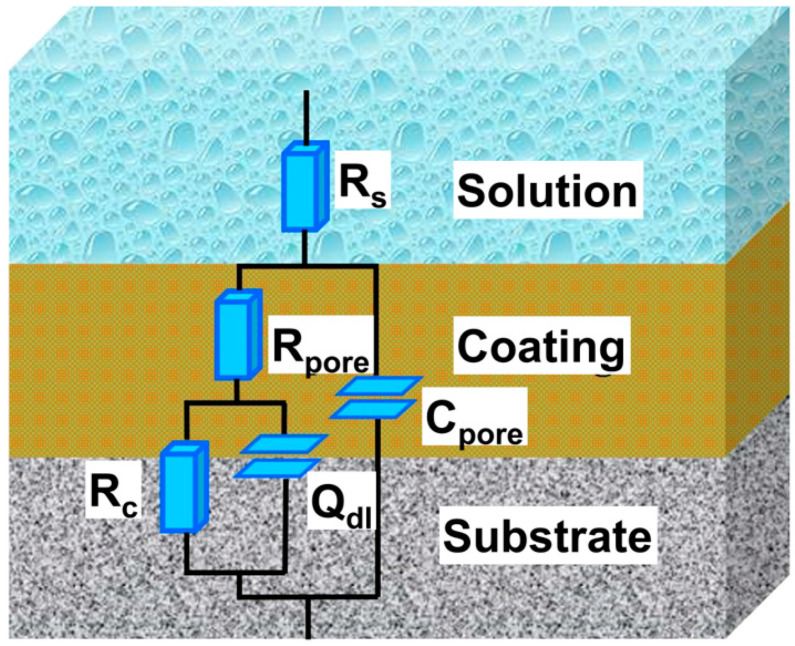
Electronic equivalent circuits employed to simulate the experimental EIS data.

The equivalent circuit was fitted to simulate the experimental EIS data, as shown in Figure 10. Since the corrosive solution would reach the coating/substrate interfaces through defects, which were formed during the preparation of the DLC coating, the equivalent circuit R_s_ (C_pore_(R_pore_(Q_dl_R_ct_))) displayed in Figure 10 was used [49,50,51,52]. Where R_s_ is the solution resistance between the sample and the reference electrode, C_pore_ and R_pore_ represent the capacitance and resistance of the coatings (or passivation coatings), and Q_dl_ and R_ct_ are the constant phase angle elements and charge transfer resistance of the double-layer capacitance, respectively [48,53].

Table 4 shows the equivalent circuit fitting results for each EIS. The fitting of the electronic equivalent circuit matched the experimental results well, as displayed in Figure 8, and the chi-square coefficient (χ^2^) was less than 10^−3^ orders of magnitude, indicating that the electronic equivalent circuit constant matched the EIS results. The charge transfer resistances (R_ct_) of Ti/DLC, TiC_x_/DLC, Ti-TiC_x_/DLC-coated 316Lss, and bare 316Lss substrates in 3.5 wt.% NaCl were 2.23 × 10^7^ Ω·cm^2^, 5.76 × 10^6^ Ω·cm^2^, 1.78 × 10^8^ Ω·cm^2^, and 1.19 × 10^6^ Ω·cm^2^, respectively (Figure 9d). As a result, the corrosion resistance of 316Lss in the 3.5 wt.% NaCl solution was greatly improved by the DLC coatings, especially the R_ct_ of the 316Lss, which was increased by more than one order of magnitude by the Ti/DLC and Ti-TiC_x_/DLC coatings. The corrosion resistance of the TiC_x_/DLC coating was inferior when compared to the previous two samples. This was primarily due to the passivation effect of Ti, which effectively sealed off pores and prevented further corrosion of the substrate. In contrast, the passivation capacity of TiC was insufficient to achieve the same level of pore sealing as Ti.

### 3.4. Electrochemical Corrosion Properties after Long-Term (63 Days) In-Situ Immersion

**Figure 11:**
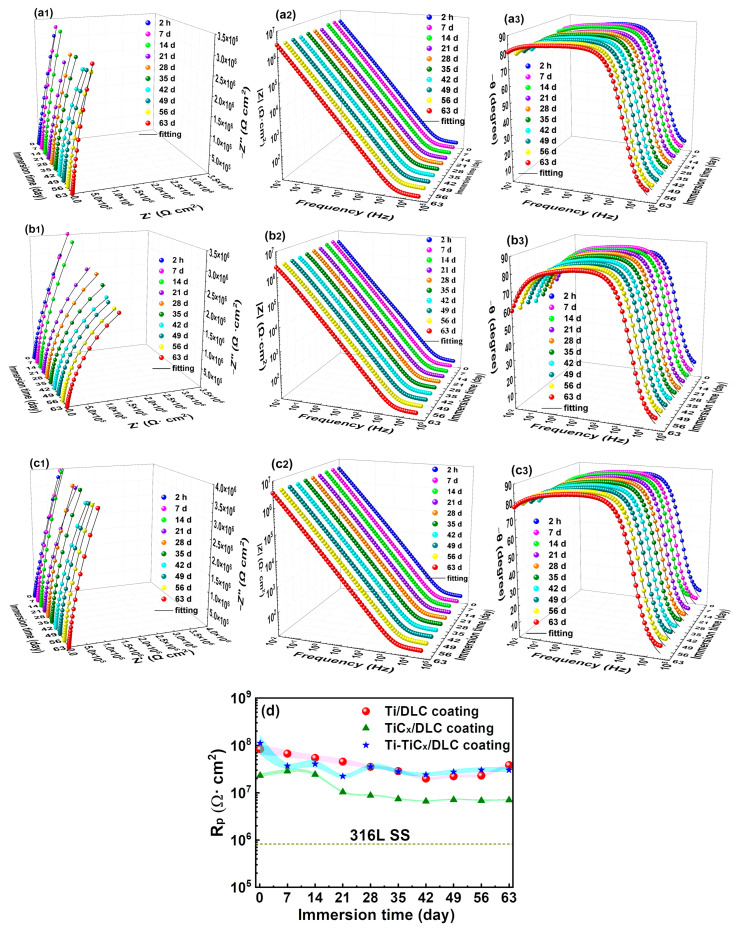
(**a1**–**c3**) EIS and (**d**) charge transfer resistance curves of three kinds of multilayer coatings and 316Lss in 3.5 wt.% NaCl.

In order to investigate their long-term corrosion resistance, the EIS of the coated samples during 63 days of immersion in 3.5 wt.% NaCl was tested, as shown in Figure 11(a1–c1). At the beginning of the immersion (1–14 days) test, the polarization resistances of the three coated samples were similar; however, the polarization resistances of TiC_x_/DLC coatings decreased significantly after immersion for 21 days but were higher than those of 316Lss, as shown in Figure 11d, while the charge transfer resistances of Ti/DLC and Ti-TiC_x_/DLC coatings decreased slightly during the whole immersion period and remained at ~3.0 × 10^7^ Ω·cm^2^ after immersion for 28 days, which inferred that all the DLC coatings protected 316Lss substrates effectively. Furthermore, after 63 days of immersion, the polarization resistances of the coated samples were still much higher than those of 316Lss, which indicated that all the DLC coatings can provide good corrosion protection for the substrates under long-term immersion conditions.

**Figure 12:**
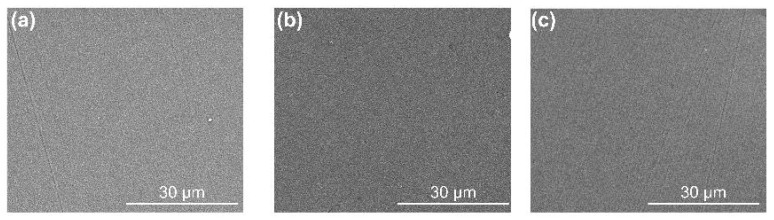
The surface SEM images of (**a**) Ti/DLC, (**b**) TiC_x_/DLC, and (**c**) Ti-TiC_x_/DLC after immersion in 3.5 wt.% NaCl for 63 days.

**Figure 13:**
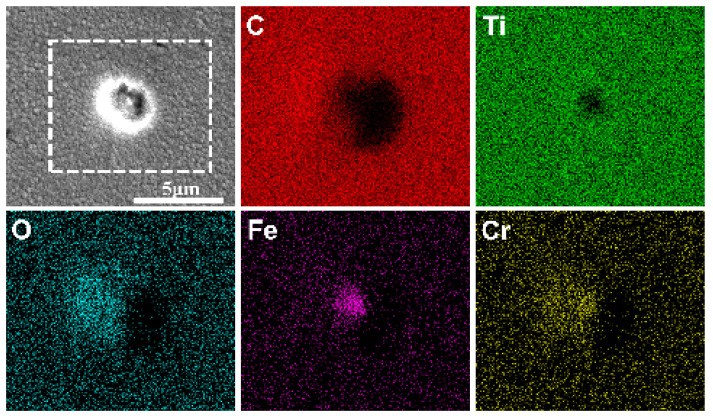
The typical local corrosion area on the Ti-TiC_x_/DLC coatings after immersion in 3.5 wt.% NaCl for 63 days and the corresponding EDS analysis results (EDS corresponds to the result in the white dotted box).

Figure 12 exhibits the surface SEM images of the three coated samples after a long-term immersion test. Their surfaces did not change significantly compared with those before immersion (Figure 3), and no obvious corrosion trace was found, while a few local corrosion areas appeared on the surface of the Ti-TiC_x_/DLC coating after immersion, resulting in the exposure of the metallic substrate, as shown in Figure 13. EDS analysis showed that Fe, Cr, and O are mainly enriched in the corrosion pit, which is characteristic of corrosion products. This defect was a penetrating defect that exposed the substrate. The evolution of this defect unfolds as follows: initially, before deposition, there was an impurity particle on the surface of the coating that would not fall during the deposition process. Subsequently, the coating developed around the shape of the impurity, forming nodular-like defects. Following this, during the cooling process after deposition, the defect detached due to thermal stress. Ultimately, a defect exposing the substrate was formed [22]. Due to the growth mechanism of this defect, the multilayer interface structure cannot avoid the growth of penetrating defects. Penetrating defects became channels for corrosion fluids to pass through, rendering them pivotal sites in the corrosion failure of multilayer coatings [23].

Those corrosion spots mostly occurred where the defects existed (Figure 9), which was already reported for DLC coatings during immersion [54].

### 3.5. Electrochemical Corrosion Properties after a 3000 h Salt Spray Test

**Figure 14:**
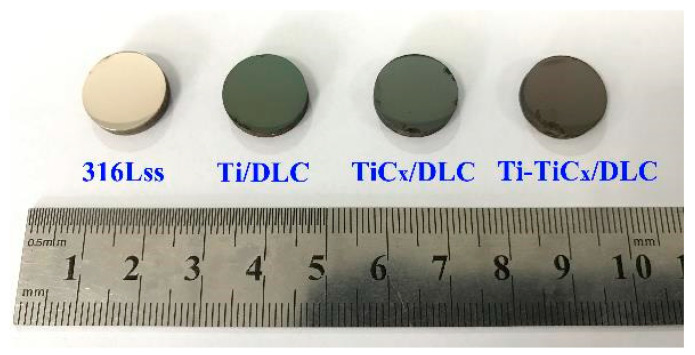
Photos of three kinds of coated samples and 316Lss after the 3000 h neutral salt spray test.

**Figure 15:**
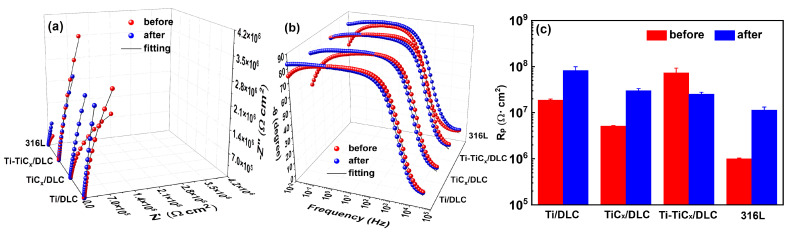
(**a**) Nyquist diagram, (**b**) Bode diagram, and (**c**) charge transfer resistances of three coated samples and 316Lss in 3.5 wt.% NaCl before and after the 3000 h neutral salt spray test.

Figure 14 shows the photos of three kinds of multilayer coatings and 316Lss after a 3000 h neutral salt spray test [55]. There was no obvious corrosion trace in the center area of 316Lss and coated 316Lss samples, and a slight corrosion trace appeared around the border of the samples due to the edge effect [56]. Figure 15 displays their electrochemical impedance spectrum and polarization resistance in 3.5 wt.% NaCl before and after the 3000 h neutral salt spray test. After the salt spray test, both the capacitive arc and the polarization resistance of 316Lss increased significantly, since the thickening of the passivation coating on the 316Lss during the salt spray test can improve its corrosion resistance [46,47,57,58,59,60,61]. Similarly, the corrosion resistance of Ti/DLC and TiC_x_/DLC also showed a certain degree of improvement, which may be due to the fact that the insoluble corrosion products at the defects of the DLC coating can block the defects and slow down the subsequent corrosion in the environment of the salt spray test [62]. In Figure 15c, after the 3000 h neutral salt spray test, the charge transfer resistances of the coated samples were higher than those of 316Lss, which indicated that all the DLC coatings could also provide excellent long-term corrosion protection for 316Lss in a salt spray corrosion environment.

## 4. Discussion

According to the electrochemical test results, at the initial stage, Ti-TiC_x_/DLC coatings displayed the best corrosion resistance because the higher number of interfaces in the Ti-TiC_x_/DLC can hinder the penetration of the corrosive media [18].

During the long-term immersion in 3.5 wt.% NaCl, the EIS test showed the polarization resistance of TiC_x_/DLC coatings decreased significantly after 21 days of immersion, while that of Ti/DLC and Ti-TiC_x_/DLC only decreased slightly and kept stable at the high value of 3.0 × 10^7^ Ω·cm^2^ during the whole immersion period. Ti-TiC_x_/DLC coatings showed more interfaces, and the single layers on either side of the interface (Fe-Cr/Ti, Ti/TiC_x_, and TiC_x_/DLC) were similar in thermal expansion coefficient and mechanical properties, which may lead to its compact interface, resulting in fewer through-holes. Usually, the through-holes and defects in DLC coatings act as fast diffusion channels for corrosive ions, such as chloride ions, to trigger the electrochemical corrosion of 316Lss and more local corrosion spots.

Different from long-term immersion, after 3000 h of salt spray, the corrosion resistance of Ti/DLC and TiC_x_/DLC coatings increased to a certain extent. Generally, the insoluble corrosion products at the defects of the DLC coating can block the defects and slow down the subsequent corrosion in the environment of the salt spray test [62]. Therefore, the enrichment of corrosion products at the defects of the DLC coating is beneficial to improving its corrosion resistance in a salt spray corrosion environment, which is different from the corrosion process during the long-term immersion since the flow of the solution would prevent the accumulation of corrosion products at the defects of the DLC coatings [54].

## 5. Conclusions

Three kinds of multilayer DLC coatings, Ti/DLC, TiC_x_/DLC, and Ti-TiC_x_/DLC, were prepared on 316Lss by linear ion beam composite DC magnetron sputtering technology. Results demonstrated that the corrosion resistance of 316Lss in 3.5 wt.% NaCl was greatly improved by the three DLC coatings. Meanwhile, EIS in-situ monitoring (63 days) and neutral salt spray (3000 h) test results showed that all three DLC coatings could provide excellent long-term corrosion protection for 316Lss, and the more interfaces and insoluble corrosion products accounted for their improved corrosion resistance, respectively. Among the three DLC coatings, Ti-TiC_x_/DLC maintained excellent corrosion resistance (R_p_ ~3.0 × 10^7^ Ωcm^2^) during the 63-day long-term immersion due to the larger number of interfaces hindering the penetration of the corrosive media. The overall corrosion resistance of the coating was stable, but corrosion failure was observed at the defects. Defects were a key factor in coating failures. This study can provide a route to designing amorphous carbon protective coatings for long-term marine applications in different environments.

## Figures and Tables

**Table 1:** Raman fitting results of three kinds of multilayer coatings.

Sample	D Peak (cm^−1^)	G Peak (cm^−1^)	G_FWHM_ (cm^−1^)	I_D_/I_G_
Ti/DLC	1368.04	1545.73	181.14	0.56
TiC_x_/DLC	1369.25	1552.84	181.73	0.59
Ti-TiC_x_/DLC	1369.80	1547.26	179.95	0.56

**Table 2:** Mechanical properties of substrates and three kinds of multilayer coatings.

Sample	H (GPa)	E (GPa)	H/E	H^3^/E^2^ (GPa)
316Lss	5.18 ± 0.16	238.46 ± 7.64	0.022	0.0024
Ti/DLC	16.44 ± 0.42	210.37 ± 4.92	0.078	0.1004
TiC_x_/DLC	22.79 ± 0.33	255.78 ± 4.57	0.089	0.1809
Ti-TiC_x_/DLC	16.53 ± 0.68	205.78 ± 8.37	0.080	0.1067

**Table 3:** Analysis results of the potentiodynamic polarization test in 3.5 wt.% NaCl.

Sample	Ti/DLC	TiC_x_/DLC	Ti-TiC_x_/DLC	316Lss
E_corr_ (V)	−0.05	−0.15	0.12	−0.19
i_pass_ (A/cm^2^)	1.12 × 10^−9^	4.27 × 10^−9^	5.68 × 10^−10^	1.86 × 10^−8^
β_a_ (mV/decade)	135.00	177.70	110.70	61.00
β_c_ (mV/decade)	87.92	66.99	244.50	46.15
R_p_ (Ω·cm^2^)	2.06 × 10^7^	4.95 × 10^6^	5.83 × 10^7^	6.13 × 10^5^
E_pit_ (V)	~1.01	~0.98	~1.00	~0.36

**Table 4:** EIS fitting results of three kinds of multilayer coatings and 316Lss in 3.5 wt.% NaCl.

Sample	Ti/DLC	TiC_x_/DLC	Ti-TiC_x_/DLC	316Lss
R_s_ (Ω·cm^2^)	18.62	20.25	23.7	22.27
C_pore_ (F·cm^−2^)	4.00 × 10^−6^	3.87 × 10^−6^	3.05 × 10^−6^	3.24 × 10^−5^
R_pore_ (Ω·cm^2^)	547,820	62,590	515,040	103,820
Q_dl_ (Ω^−1^·cm^−2^·s^n^)	3.43 × 10^−7^	8.255 × 10^−7^	3.83 × 10^−7^	2.48 × 10^−6^
n_dl_	0.651	0.639	0.65	0.50
R_ct_ (Ω·cm^2^)	2.23 × 10^7^	5.76 × 10^6^	1.78 × 10^8^	1.19 × 10^6^
χ^2^	3.22 × 10^−4^	2.82 × 10^−4^	9.37 × 10^−4^	4.94 × 10^−4^

## Data Availability

Data are contained within the article.
